# The Medial Longitudinal Fasciculus and Internuclear Opthalmoparesis: There’s More Than Meets the Eye

**DOI:** 10.7759/cureus.9959

**Published:** 2020-08-23

**Authors:** Peter Fiester, Dinesh Rao, Erik Soule, Sonia Andreou, Dalys Haymes

**Affiliations:** 1 Neuroradiology, University of Florida Health, Jacksonville, USA; 2 Interventional Radiology, University of Florida College of Medicine, Jacksonville, USA; 3 Surgery, University of Florida College of Medicine, Jacksonville, USA

**Keywords:** medial longitudinal fasciculus, internuclear opthalmoplegia, cerebrovascular accident, multiple sclerosis, demyelinating disease, magnetic resonance imaging

## Abstract

Background and purpose

The classic sign of a lesion in the medial longitudinal fasciculus is internuclear opthalmoplegia. However, clinical presentation may vary depending on the type of pathology and the lesion location. The purpose of this study was to identify and classify the different lesions of the medial longitudinal fasciculus on MRI and review their clinical presentations. We also offer an overview of the pertinent imaging anatomy of the medial longitudinal fasciculus.

Materials and methods

Patients with an abnormality affecting the medial longitudinal fasciculus were identified retrospectively using the keyword ‘medial longitudinal fasciculus’ included in radiology reports between 2010 and 2018 using the Nuance mPower software (Nuance Communications, Burlington, MA). The brain MRI examinations of these patients were reviewed by two neuroradiolgists. Detailed description of lesion location within the medial longitudinal fasciculus, pathology type, additional lesions, and clinical presentations were recorded along with pertinent demographic information.

Results

Five men and four women were identified with lesions in the medial longitudinal fasciculus on brain MRI. Five patients demonstrated demyelination in the medial longitudinal fasiculus and four patients demonstrated findings of an acute cerebrovascular accident. Two-thirds of medial longitudinal fasiculus lesions were located in the upper, mid, or lower pons with the remaining located in the midbrain. Of the patients presenting with a cerebrovascular accident, there was little to no additional evidence of acute stroke elsewhere in the brain. All patients were clinically symptomatic with 89% of patients demonstrating extraocular muscle dysfunction at presentation and 78% of patients experiencing dizziness. Additional symptoms included headache, weakness, and gait instability.

Conclusions

Lesions involving the medial longitudinal fasiculus may not always present with the classic sign of internuclear opthalmoplegia. Variations in lesion location may result in diplopia rather than internuclear opthalmoplegia, and additional brain lesions may produce clinical symptoms that confound extraocular muscle dysfunction. Lesions affecting the often-overlooked vestibular and otolithic reflexes, which run in the medial longitudinal fasiculus, may result in dizziness/weakness and mask the classic internuclear opthalmoplegia symptoms. The radiologist should carefully inspect the medial longitudinal fasiculus in all patients regardless of the supportive clinical history of extraocular muscle dysfunction since symptoms may be more general than classically described.

## Introduction

The medial longitudinal fasciculus (MLF) is a paired, highly specialized and heavily myelinated nerve bundle traveling in a craniocaudal direction near the midline within the tegmentum of the midbrain and dorsal pons immediately ventral to the cerebral aqueduct and fourth ventricle. Functionally, the MLF carries excitatory and inhibitory axonal nerve fibers from the frontal eye field, paramedian pontine reticular formation (PPRF), and cranial nerves (CN) III, IV, and VI. These fibers produce agonist and antagonist extraocular muscle (EOM) contraction for saccadic eye movement (eye movement allowing for change in fixation point) and smooth pursuit (eye movement allowing for following a moving object). Vestibular-ocular nerve fibers traveling from CN VIII include both a semicircular canal and otolith-mediated oculomotor reflex, running within the MLF to help coordinate balance and linear acceleration, respectively [1].

Depending on the location of a lesion affecting the heavily myelinated tracts of the MLF, specific clinical signs are produced. These signs are clinically divided into four categories of disease: (1) internuclear opthalmoplegia (INO), (2) INO and trochlear syndrome, (3) INO and one and a half syndrome, and (4) wall-eyed bilateral INO syndrome [2]. A lesion affecting the MLF that results in classic INO presents as a lack of adduction of the ipsilateral eye with preservation of abduction of the contralateral eye. This is best appreciated with saccadic eye movement on clinical exam [3]. INO and trochlear syndrome results from a lesion affecting the MLF at the caudal midbrain as well as the adjacent ipsilateral trochlear nucleus. This presents as INO with a contralateral hyperdeviation because the loss of trochlear innervation affects the contralateral superior oblique muscle. A lesion affecting the MLF at the dorsal pontine tegmentum and adjacent ipsilateral abducens nucleus or PPRF results in INO and one and a half syndrome, INO in one direction and lateral gaze palsy in the other direction. Wall-eyed bilateral INO syndrome is a failure of convergence resulting from a lesion of the bilateral MLF and presenting clinically as divergence of the eyes ('wall-eyed'). We define these clinical entities separately from isolated lesions of the oculomotor nuclei, which may cause vertical or horizontal diplopia as well as ptosis. Lesions of the abducens nuclei and/or PPRF may alternatively present with an isolated lateral gaze palsy.

Finally, the MLF transmits the axonal connections responsible for compensatory eye movement for angular motion of the head between CN VIII and the lateral, anterior, and posterior semicircular canals and the EOM nuclei [4-7]. For example, horizontal head motion stimulates the lateral semicircular canal, which in turn excites the ipsilateral CN VIII nerve and nuclei. Axonal projections then extend from the medial vestibular nucleus within the MLF to the contralateral CN VI nuclei to excite the lateral rectus muscle, while interneurons from the abducens nuclei via the MLF excite the medial rectus subnuclei for CN III for compensatory lateral eye movement. Similar pathways exist for both the anterior and posterior semicircular canals [8,9]. Likewise, the otolith organs detect linear acceleration and transmit acceleration stimuli via the vestibular nuclei through the MLF to the EOM nuclei; however, this pathway is less well understood [10].

To investigate the degree of correlation between lesion location on MRI and clinical presentation, we reviewed the MRIs of patients with MLF lesions and their physical signs and symptoms. We compared the lesion location in the brainstem and the type and extent of pathology to patients’ demographic information, presenting clinical signs and symptoms, and physical exam findings. 

## Materials and methods

A waiver of informed consent was granted by the University of Florida Health Jacksonville institutional review board (IRB) chairman to retrospectively evaluate the imaging and clinical findings of patients who suffered a lesion in the MLF. Nine adult patients with a confirmed lesion in the MLF on brain MRI were identified retrospectively by a keyword search of radiology reports using Nuance mPower software (Nuance Communications, Burlington, MA) between January 2013 and July 2018 using the keyword ‘medial longitudinal fasciculus’.

MRI exams were performed using the standard departmental protocols. MRI studies were performed on a 1.5 or 3.0 Tesla magnet with a head and neck coil (Siemens, Munich, Germany). Slice thickness was 4 mm and axial T1, T2, fluid-attenuated inversion recovery (FLAIR), diffusion-weighted imaging (DWI), gradient recall echo (GRE), and susceptibility-weighted imaging (SWI) sequences were reviewed as well as postcontrast axial T1 and magnetization prepared rapid gradient echo (MP-RAGE) sequences if IV contrast was administered. All MRI exams were reviewed by two experienced certificate of additional qualification certified neuroradiologists in consensus.

The MLF was considered abnormal when the T2 signal within the dorsal midbrain and pons near the midline was higher than the adjacent brain parenchyma. Furthermore, the location of the MLF lesion was recorded and divided into ‘superior tegmentum’ (superior half of the midbrain), ‘inferior tegmentum’ (inferior half of the midbrain), ‘superior pontine’ (superior half of pons), ‘mid pontine’ (level of the fastigium of fourth ventricle), and ‘inferior pontine’ (inferior half of pons). The type of pathology (e.g. demyelination, stroke) was recorded along with the presence of additional lesions. For demyelination, the presence and absence of plaques within the cerebral hemispheres, cerebellum, and brainstem (other than the MLF) were recorded, and the number of plaques was categorized as mild, moderate, and severe. For stroke, the presence and absence of other areas of stroke and location were recorded. Electronic patient records were reviewed for the following: (1) age and sex of patient, (2) clinical symptoms, (3) physical exam findings, and (4) clinical management.

## Results

Nine patients were identified with a lesion in the MLF, including five male patients and four female patients ranging in age from 30 to 75 years with a mean age of 52 years. Two-thirds of our patients had an MLF lesion in the pons (one patient in superior pons, four patients in mid pons, and one patient in inferior pons) and one-third of our patients had an MLF lesion in the midbrain (one patient in superior midbrain and two patients in inferior midbrain). Pathology affecting the MLF included cerebrovascular accident (CVA) (four patients) and primary demyelination (five patients). In the patients with a CVA in the MLF, 75% exhibited no findings of CVA elsewhere in the brain, whereas all patients with primary demyelination in the MLF exhibited additional demyelinating plaques in the brain (Table 1).

**Table 1:** Type of pathology, location, presence of additional lesions, and neurological clinical signs and symptoms were recorded for patients with a lesion in the medial longitudinal fasciculus on brain MRI. CVA: cerebrovascular accident, INO: internuclear opthalmoplegia

Number	Sex/age	Pathology	Location	Additional lesions	Symptoms/signs
1	M/72	CVA	Right upper pons	Minimal punctate CVA	Diplopia; dizziness; right INO; left nystagmus
2	M/39	Demyelination	Right mid pons	Minimal periventricular plaques	Diplopia; dizziness; bilateral INO
3	M/30	Demyelination	Right mid pons	Minimal periventricular plaques	Diplopia; dizziness
4	F/52	CVA	Right mid pons	None	Diplopia; impaired right eye adduction
5	M/75	CVA	Right upper midbrain	None	Dizziness; right INO; left nystagmus
6	F/54	CVA	Right upper midbrain	None	Dizziness; headache; right INO; left nystagmus
7	M/52	Demyelination	Bilateral inferior midbrain	Moderate periventricular plaques	Weakness; dizziness
8	F/39	Demyelination	Left inferior pons	Moderate periventricular plaques	Gait instability; right leg weakness; right ptosis
9	F/56	Demyelination	Bilateral mid pons	Minimal periventricular plaques	Dizziness; right nystagmus

Upon review of the neurology clinic note preceding the brain MRI, all patients with a lesion in the MLF were clinically symptomatic. Seven patients exhibited EOM dysfunction, with four patients exhibiting the clinical findings of internuclear opthalmoparesis and three patients exhibiting diplopia without nystagmus. In the patients with signs of INO, half of those patients had a lesion in the midbrain and half had a lesion in the pons.

The most common reported non-EOM-related clinical symptom in our patients was dizziness (78%) with other less commonly reported clinical symptoms, including headache, weakness, and gait instability.

## Discussion

**Figure 1:**
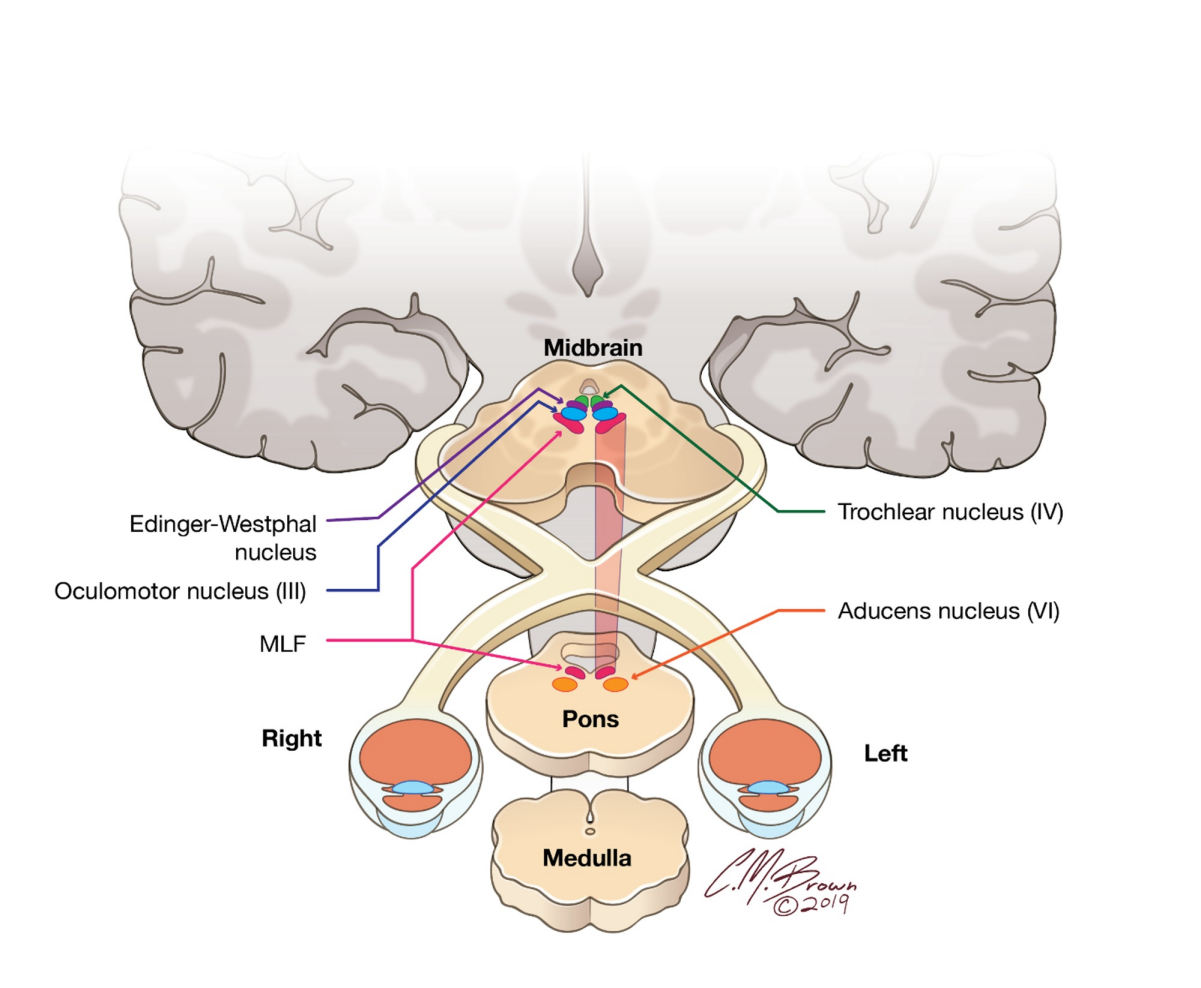
Illustration demonstrating the anatomic location of the medial longitudinal fasciculus (MLF) running in the midline in the dorsal midbrain and pons and its relationship to the extraocular muscle nuclei (cranial nerves III, IV, and VI). Illustrations provided by and with the permission of Christopher Brown.

Internuclear opthalmoparesis is the clinical finding most commonly associated with lesions affecting the MLF on brain MRI (Figure 1). 

**Figure 2:**
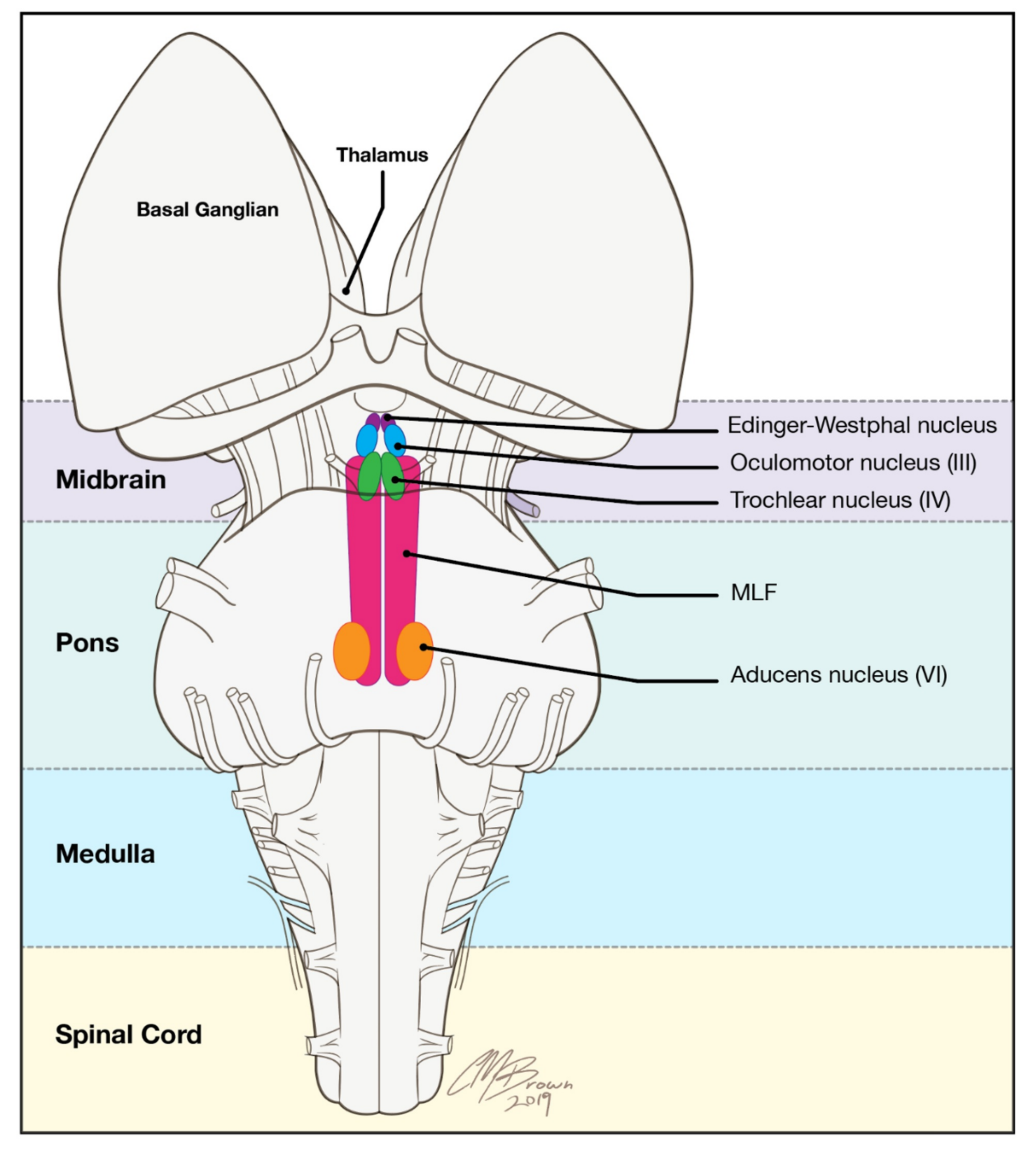
The medial longitudinal fasciculus (MLF) is a specialized and heavily myelinated nerve bundle adjacent to the cranial nerve III and IV nuclei in the midbrain. It extends in a craniocaudad dimension to the level of the cranial nerve VI nuclei in the inferior and dorsal pons. Illustrations provided by and with the permission of Christopher Brown.

Classic INO is the result of a brain lesion confined to the MLF that also spares the trochlear nuclei, PPRF, and contralateral MLF (Figure 2). 

**Figure 3:**
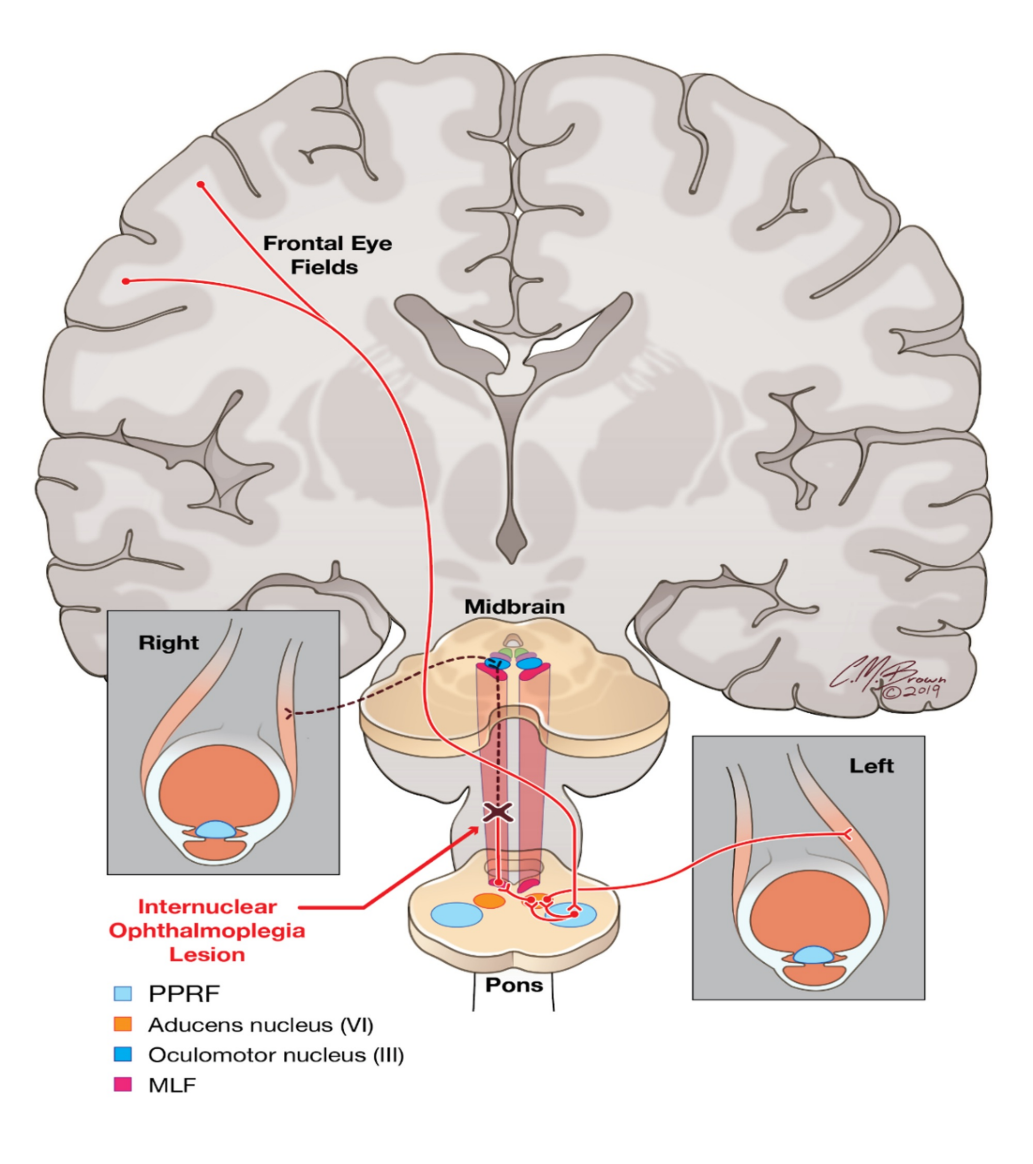
Neurological pathway for synchronized, horizontal conjugate gaze. Input from the frontal eye field activates the paramedian pontine reticular formation (PPRF), which in turn transmits two signals via the CN VI nuclei. One signal activates the ipsilateral lateral rectus muscle, while another signal travels in the contralateral MLF to activate the contralateral CN III nuclei and the medial rectus muscle. A lesion in the MLF may cause INO (preserved abduction of the contralateral eye with nystagmus and weakened adduction of the ipsilateral eye). Illustrations provided by and with the permission of Christopher Brown. CN: cranial nerve, MLF: medial longitudinal fasiculus, INO: internuclear opthalmoplegia

Following an excitatory impulse from the contralateral frontal eye field (cerebral cortex), the PPRF is activated. The PPRF then relays two signals via the ipsilateral abducens nucleus: one signal activates the abducens nucleus to cause contraction of the ipsilateral lateral rectus muscle and another signal runs in the contralateral MLF to the oculomotor nucleus to activate the medial rectus muscle (Figure 3). 

Thus, a lesion confined to the MLF disrupts the signal to the contralateral oculomotor nucleus and its activation of the medial rectus muscle. Clinically, this results in preserved abduction of the contralateral eye with nystagmus, and weak or absent adduction of the ipsilateral eye. 

Our retrospective case review investigated the degree of correlation between an MLF lesion on brain MRI and classic INO based on the neurology clinic note of an experienced neurologist at a tertiary academic center. In our patient population, only four of nine patients with a lesion in the MLF presented with the classic signs of INO. These four patients demonstrated an MLF lesion between the midbrain and mid pons at the level of the fastigium of the fourth ventricle (Table 2).

**Table 2:** Overview of internuclear opthalmoplegia and related syndromes.

	Signs/symptoms	Lesion location	Involved structures	Ocular muscles denervated
Internuclear opthalmoplegia	Lack of adduction of the ipsilateral eye with preservation of abduction of the contralateral eye, contralateral nystagmus, horizontal diplopia, preserved convergence	Midline pons/midbrain	Medial longitudinal fasciculus	Ipsilateral medial rectus
Internuclear opthalmoplegia and trochlear syndrome	Lack of adduction of the ipsilateral eye with a contralateral hyperdeviation, preserved convergence	Caudal midbrain	Medial longitudinal fasciculus and ipsilateral trochlear nucleus	Ipsilateral medial rectus and contralateral superior oblique
Internuclear opthalmoplegia and one and a half syndrome	Lack of adduction of the contralateral eye and horizontal gaze palsy of the ipsilateral eye, preserved convergence	Dorsal pons	Medial longitudinal fasciculus and ipsilateral abducens nucleus or paramedian pontine reticular formation	Contralateral medial rectus and ipsilateral medial and lateral recti
Wall-eyed bilateral internuclear opthalmoplegia syndrome	Failure of convergence, extropia	Midline pons/midbrain	Bilateral medial longitudinal fasciculus	Bilateral medial recti

Five patients with an MLF lesion on brain MRI did not exhibit the classic findings of INO on exam. Other symptoms included a more general diplopia in addition to dizziness, gait instability, weakness, and headache. These findings suggest that patient symptoms may be confounded by other sites of intracranial pathology (which was more common in the patients with multiple sclerosis and primary demyelination), involvement of the MLF-mediated vestibular and otolithic pathways, and/or inconsistencies in physical exam evaluation such as not utilizing specific eye movement tracking techniques.

**Figure 4:**
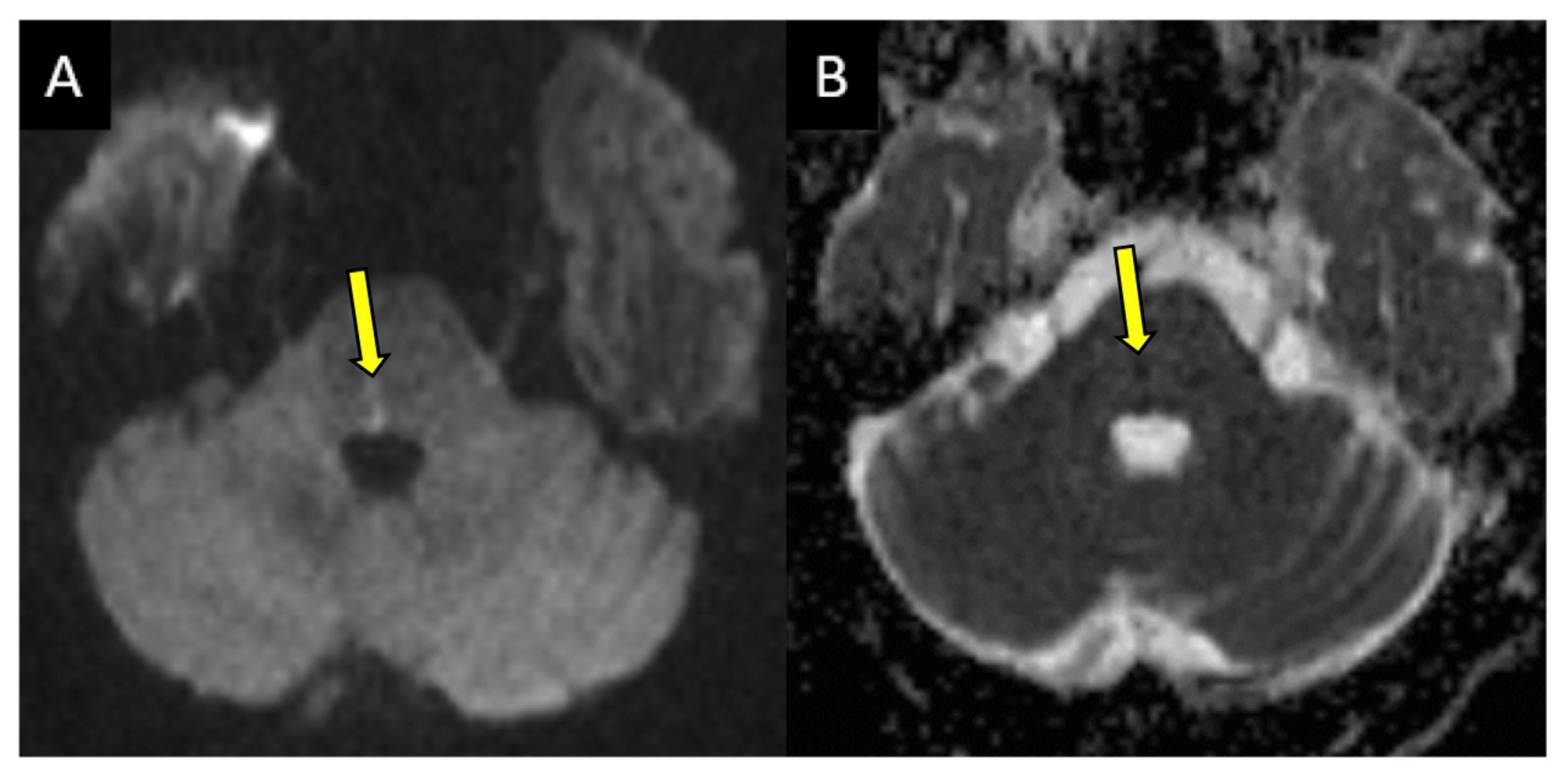
A 72-year-old male presented with diplopia, dizziness, right INO, and left nystagmus. (A) Diffusion-weighted sequence. (B) Corresponding ADC map demonstrating focal water restriction in the right dorsal pons consistent with a small acute infarct (arrows). INO: internuclear opthalmoplegia, ADC: apparent diffusion coefficient

The causative pathology in our patients is consistent with the previously published literature, with five patients presenting with primary demyelination and four patients presenting with CVA [11-13]. Approximately 75% of patients who presented with CVA demonstrated isolated ischemic infarcts (two in the midbrain and one in the upper pons) without acute ischemic change elsewhere in the brain (Figure 4). 

The MLF in the dorsal tegmentum of the midbrain is supplied by small perforating branches of the P2 segment of the posterior cerebral artery as it passes through the ambient cistern [14]. Isolated stroke in this location is a relatively rare finding [15]. The MLF in the dorsal pons is supplied by small, perforating paramedian arteries that arise from the basilar artery. Small embolic events are likely responsible for isolated CVA affecting the MLF in these locations.

**Figure 5:**
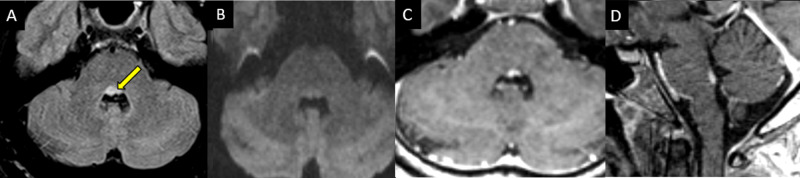
A 39-year-old male with a known clinical history of multiple sclerosis presented with bilateral INO, diplopia, and dizziness. (A) Axial FLAIR sequence demonstrates a lesion in the dorsal pons in the medial longitudinal fasciculus (arrow). (B) Axial DWI sequence demonstrates no restricted diffusion to suggest an acute infarct. (C, D) Axial and sagittal postcontrast T1 axial images demonstrate solid enhancement in this location. INO: internuclear opthalmoplegia, FLAIR: fluid-attenuated inversion recovery, DWI: diffusion-weighted imaging

Amongst the five patients with a primary demyelinating plaque in the MLF, only one patient exhibited the classic findings of INO. This was also the only patient with an MLF plaque that enhanced following the administration of intravenous gadolinium, which suggested active and ongoing inflammation and demyelination (Figure 5).

**Figure 6:**
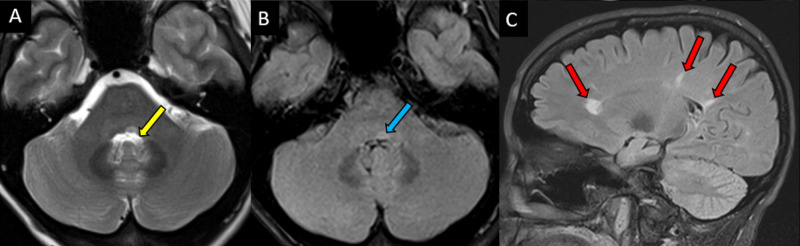
A 52-year-old male with a known history of multiple sclerosis presented with dizziness and weakness, but reported no extraocular muscle dysfunction. (A, B) Axial T2-weighted (top left) and FLAIR sequence (top right) demonstrating a small, focal demyelinating plaque in left dorsal pons (yellow and blue arrows). (C) Sagittal FLAIR sequence demonstrating the characteristic periventricular appearance of whiter matter plaques. FLAIR: fluid-attenuated inversion recovery

These findings may account for this particular patient's symptoms at the time, in contrast to the other four patients without INO symptoms (Figure 6). 

Furthermore, all patients with a demyelinating plaque in the MLF demonstrated demyelinating plaques elsewhere in the brain. Neurological findings were more vague in these patients and included dizziness, weakness, headache, gait instability, and ptosis. The presence of additional plaques may have confounded patient clinical signs and symptoms and subsequently masked the findings of INO on physical exam. 

The most common clinical symptom shared among all patients with CVA or demyelinating plaque affecting the MLF was dizziness (78% of patients). While dizziness is not a classic symptom associated with an MLF lesion, it can occur related to disruption of the vestibular ocular reflexes that also run in the MLF. The vestibular-ocular nerve fibers traveling from CN VIII include both a semicircular and otolith-mediated oculomotor reflex that run within the MLF to help coordinate balance and linear acceleration, respectively. Thus, an MLF lesion may disrupt this reflex resulting in dizziness or gait instability. Dizziness is a very common symptom resulting in the acquisition of an MRI for further evaluation. Based on these findings, we postulate that patients with the clinical presentation of dizziness may benefit from small field-of-view, T2-weighted, and diffusion-weighted imaging of the brainstem and cerebellum to further evaluate for subtle abnormalities of the MLF.

## Conclusions

Lesions affecting the MLF on brain MRI may not always present with the classic history of INO, and thus may be overlooked by the radiologist, especially considering the potentially small size of a lesion and lack of conspicuity on non-T2-weighted sequences. Our review suggests that the clinical signs and symptoms of a lesion in the MLF may be broader than previously thought with dizziness being a more common presenting symptom than classic INO on physical exam. The radiologist should carefully inspect the MLF for pathology in all patients, regardless of supportive clinical history, since symptoms may be more variable than classically described.

## References

[1] Bae YJ, Kim JH, Choi BS, Jung C, Kim E (2013). Brainstem pathways for horizontal eye movement: pathologic correlation with MR imaging. RadioGraphics.

[2] Frohman TC, Galetta S, Fox R (2008). Pearls & Oy-sters: the medial longitudinal fasciculus in ocular motor physiology. Neurology.

[3] Chen CM, Lin SH (2007). Wall-eyed bilateral internuclear ophthalmoplegia from lesions at different levels in the brainstem. J Neuroophthalmol.

[4] Frohman EM, Zhang H, Kramer PD, Fleckenstein J, Hawker K, Racke MK, Frohman TC (2001). MRI characteristics of the MLF in MS patients with chronic internuclear ophthalmoparesis. Neurology.

[5] Baloh RW, Yee RD, Honrubia V (1978). Internuclear ophthalmoplegia. I. Saccades and dissociated nystagmus. Arch Neurol.

[6] Meienberg O, Muri R, Rabineau P (1986). Clinical and oculographic examinations of saccadic eye movements in the diagnosis of multiple sclerosis. Arch Neurol.

[7] Atlas SW, Grossman RI, Savino PJ (1987). Internuclear ophthalmoplegia: MR-anatomic correlation. AJNR Am J Neuroradiolol.

[8] Carpenter MB (1988). Vestibular nuclei: afferent and efferent projections. Prog Brain Res.

[9] Ranalli PJ, Sharpe JA (1988). Vertical vestibulo-ocular reflex, smooth pursuit and eye-head tracking dysfunction in internuclear ophthalmoplegia. Brain.

[10] Cremer PD, Migliaccio AA, Halmagyi GM, Curthoys IS (1999). Vestibulo-ocular reflex pathways in internuclear ophthalmoplegia. Ann Neurol.

[11] Bolanos I, Lozano D, Cantu C (2004). Internuclear ophthalmoplegia: causes and long-term follow-up in 65 patients. Acta Neurol Scand.

[12] Kim JS (2004). Internuclear ophthalmoplegia as an isolated or predominant symptom of brainstem infarction. Neurology.

[13] Kupfer C, Cogan DG (1966). Unilateral internuclear ophthalmoplegia. A clinicopathological case report. Arch Ophthalmol.

[14] Kim SM, Kim HK, Lee HJ (2014). Medial longitudinal fasciculus on MRI in a patient with internuclear ophthalmoparesis: a case report. J Korean Soc Magn Reson Med.

[15] Kobayashi Z, Iizuka M, Tomimitsu H, Shintani S (2014). Isolated medial longitudinal fasciculus syndrome due to small midbrain infarction. Neurol Clin Neurosci.

